# Cause rare d’hypoplasie sévère: la transformation gélatineuse de la moelle osseuse

**Published:** 2010-08-21

**Authors:** Mohamad Oukabli, Selim Jennane, Hafsa Chahdi, Issam Rharrassi, Kamal Doghmi, Mohamed Mikdame, Abderrahane Albouzidi

**Affiliations:** 1 Service d’anatomie pathologique, Hôpital militaire d’instruction Mohamed V Rabat, Maroc; 2 Service d’Hématologie Clinique, Hôpital militaire d’instruction Mohamed V Rabat, Maroc

**Keywords:** Hypoplasie, pancytopénie, moelle osseuse, transformation gélatineuse

## Abstract

Nous rapportons chez une patiente de 17 ans un cas rare d’hypoplasie sévère avec transformation gélatineuse de la moelle osseuse dont l’étiologie spécifique reste mystérieuse malgré un bilan diagnostique et étiologique assez détaillé. La transformation gélatineuse (encore appelée atrophie séreuse, ou moelle de déprivation) est caractérisée par l’association d’une hypoplasie médullaire et d’une infiltration interstitielle par une substance gélatineuse amorphe formée de mucopolysaccharides acides. Elle s’accompagne d’anomalies sanguines périphériques qui sont variables, souvent modérées et non corrélées à la gravité de l’atteinte centrale. Sa pathogénie, encore mal connue, est très certainement liée à la dénutrition chronique et les pathologies associées à une cachexie.

## Introduction

Dénommée "atrophie séreuse médullaire" ou "moelle de privation" [1-5], la transformation gélatineuse de la moelle osseuse est une lésion histologique rare ; elle est classiquement rencontrée dans un contexte de dénutrition. Ses étiologies sont fréquentes. Le pronostic de ces lésions non spécifiques et d’étiopathologie variable dépend du diagnostic étiologique [2].

Nous rapportons chez une patiente de 17 ans un cas rare d’hypoplasie sévère avec transformation gélatineuse de la moelle osseuse dont l’étiologie spécifique reste mystérieuse malgré un bilan diagnostique et étiologique assez détaillé.

## Patient et observation

Il s’agit d’une patiente de 17 ans, dont les antécédents familiaux révélaient un frère décédé à l’âge d’un mois de cause inexpliquée, un cousin décédé à l’âge de 14 ans par aplasie médullaire. Le début remontait à 5 mois par l’installation brutale d’un syndrome d’insuffisance médullaire fait d’un syndrome anémique (pâleur cutanéo-muqueuse, asthénie, vertige), un syndrome hémorragique fait de gingivorragie, méno-métrorragie et moelena et un syndrome infectieux fait de fièvre non chiffrée avec un amaigrissement chiffré à 5 kg en 4 mois. Ce qui avait motivé une hospitalisation dans un hôpital régional où un bilan révélait une pancytopénie avec une aplasie médullaire d’allure idiopathique à la biopsie ostéomédullaire. Puis était adressée à notre formation pour complément de prise en charge.

L’examen d’admission trouvait une patiente en mauvais état général, une pâleur cutanéo-muqueuse, une fièvre à 40°C avec frissons, un retard staturo pondérale (1,50m, 35kg), un visage triangulaire, flexion interne congénitale des 2 articulations inter phalangiennes distales des 2 annulaires. Les 2 genoux et les 2 chevilles étaient tuméfiés, chauds, sans signes inflammatoires en regard, avec 2 taches café au lait congénitales au niveau du torse et du dos d’environ 2 cm. Une gingivorragie de faible abondance et des taches purpuriques pétéchiales au niveau des membres. Le bilan biologique révélait une pancytopénie avec un taux d’hémoglobine (Hb) à 5,7; globule blanc (GB) à 1200; PNN (polynuclear neutrophil count) à 300 et PQ à 15000; C-reactive protein (CRP) à 120mg/l; l’ionogramme, fonctions rénale et hépatique normales. Il existait une hypo protidémie (55g/l), hypo albuminémie, hypocholestérolémie avec absence d’anticorps anti endomesium et anti gliadine. Les sérologies HIV, Hépatite C, Hépatite B était négatives et le caryotype normale. L’examen cytobactériologique des urines (ECBU) était stérile, la radiographie des poumons était normale et l’hémoculture négative. La relecture de la biopsie ostéomédullaire révélait un aspect typique de transformation gélatineuse de la moelle hématopoïétique. Le tissu médullaire était presque en totalité remplacé par une substance myxoïde éosinophile, pâle avec parfois de rares éléments hématopoïétiques ( 1 et 2), colorée par le bleu alcian pH 2,5 ( 3). Le bilan d’hémostase révélait un TP (taux de prothrombine) normale et TCA (Temps de Céphaline + activateur) allongé, un indice de Rosner de 0,45 et le dosage des facteurs de coagulation normale avec présence d’un anticorps antiphospholipide.

Le bilan immunologique demandé devant la polyarthrite inflammatoire était négatif (Ac anti peptides cycliques citrullinés, Auto anticorps anti J01, Auto anticorps anti SCL70, facteurs rhumatoïde, anticorps antinucléaires, anticorps anti DNA natif, anti antigène nucléaire soluble SSA et SSB et RNP, anticorps anti-Sm). La densité osseuse était basse à l’ostéo densitométrie. Sur le plan thérapeutique la patiente a été mise sous: antibiothérapie: claforan genta, ciclosporine à la dose de 8 mg/kg/j, Support transfusionnelle agressif en plaquette et en rouge, Hydratation abondante et les morphiniques.

L’évolution était marquée par la persistance de la fièvre avec passage au tienam, vancomycine et amikacine. Devant la non amélioration du syndrome infectieux, une hémoculture sur sang périphérique était revenu en faveur d’un bêta-lactamase à spectre élargie (BLSE) multi résistant, sensible au tienam, amiklin et colymicine.

Sur le plan hématologique, les besoins transfusionnels restaient importants avec des besoins d’environ 2 culots globulaires tous les 7 jours et des besoins plaquettaires de 10 CPS un jour sur 3 en moyenne. A 8mg/k/j de ciclosporine la patiente avait développé une cytolyse à 8 fois la normale et une cholestase à 4 fois la normale, d’où la diminution des doses à 7mg/kg/j. Vu la difficulté de la prise des voies veineuses périphériques, une chambre implantable était mise en place.

Devant la polyarthrite inflammatoire, la persistance de la fièvre malgré une antibiothérapie large, des bolus de corticoïde étaient délivrés (120 mg de solumédrol par jour) mais sans réponse significative. La persistance des signes infectieux et la survenue d’une chute tensionelle une Echocœur montrait la présence d’un thrombus accolé à l’extrémité distale du PAC au niveau de l’oreillette droite avec une fraction d’éjection basse qui évoquait une myopathie infectieuse. La chambre implantable était retirée. Une semaine après, la patiente était décédée dans un tableau de choc septique avec défaillance multiviscérale, le tout sans étiologie évidente.

**1:**
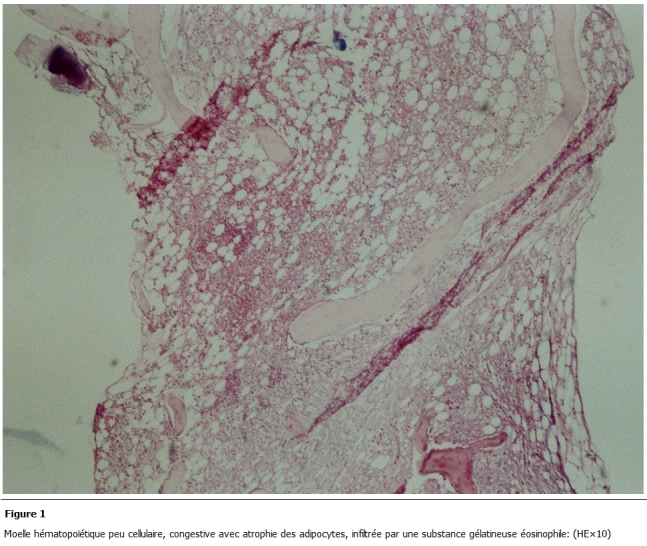
Moelle hématopoïétique peu cellulaire, congestive avec atrophie des adipocytes, infiltrée par une substance gélatineuse éosinophile. (HE×10)

**2:**
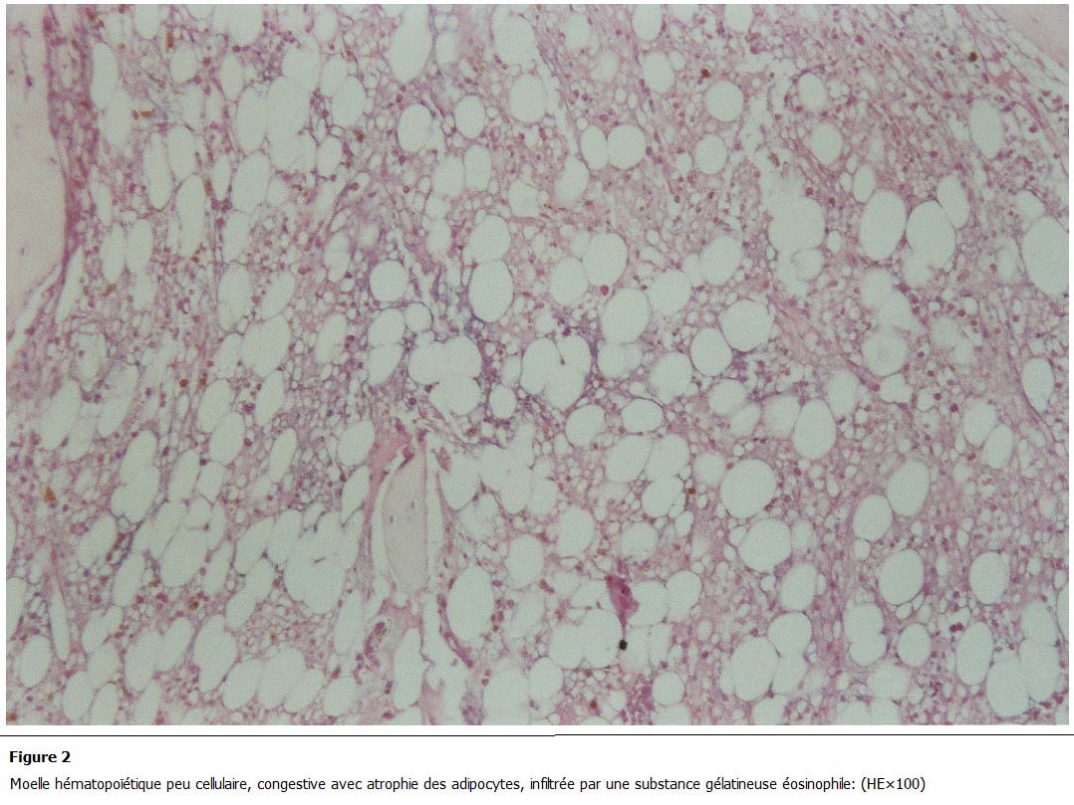
Moelle hématopoïétique peu cellulaire, congestive avec atrophie des adipocytes, infiltrée par une substance gélatineuse éosinophile. (HE×100)

**3:**
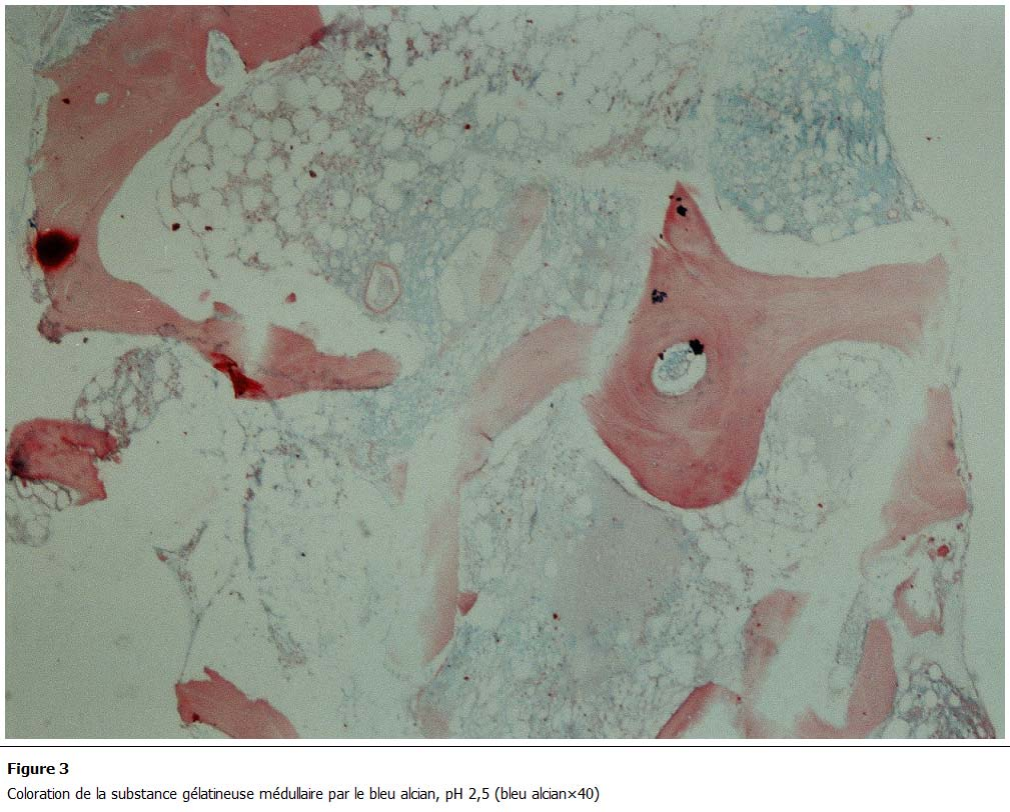
Coloration de la substance gélatineuse médullaire par le bleu alcian, pH 2,5 (bleu alcian×40).

## Discussion

La transformation gélatineuse de la moelle hématopoïétique est une affection rare, décrite à la fin du XIXe siècle. Sa pathogénie reste mal connue mais elle est caractérisée histologiquement par une hypoplasie des trois lignées hématopoïétiques avec une atrophie des adipocytes médullaires associée à l’accumulation d’une substance gélatineuse constituée de mucopolysaccharides et d’acide hyaluronique, colorée par le bleu alcian [1-6].

La transformation gélatineuse de la moelle se traduit par des perturbations du bilan hématologique, souvent à type d’anémie ou de pancytopénie comme dans notre observation. Une anémie arégénérative, normochrome et normocytaire doit faire envisager la pratique d’un myélogramme et si celui-ci n’est pas contributif, la réalisation d’une biopsie ostéomédullaire [7].

Les formes les plus sévères de transformation gélatineuse médullaire sont observées chez les patients jeunes comme notre patiente qui était âgée de 17 ans et qui présentait une hypoplasie sévère [1-3].

En imagerie les foyers de transformation gélatineuse sont caractérisés au niveau des os atteints par un hyposignal en séquence T1 et un hypersignal en séquence T2 à l’imagerie par résonance magnétique (IRM) [3], cependant elle n’avait pas d’indication chez notre patiente.

La transformation gélatineuse de la moelle n’est pas une maladie spécifique mais le symptôme d’une pathologie systémique sévère qui reste mystérieuse chez notre patiente malgré un bilan diagnostique et étiologique assez détaillé [1-6]. Les causes sont variées mais la transformation gélatineuse de la moelle survient généralement dans le cadre d’une cachexie chez un patient ayant une maladie systémique (lupus), une septicémie, une anorexie mentale, un sida, une pathologie cancéreuse, un alcoolisme chronique, une insuffisance cardiaque sévère, une maladie métabolique (diabète, hypothyroïdie) ou une défaillance multi viscérale chez des malades hospitalisés en réanimation [1-5].

Les observations de transformation gélatineuse de la moelle survenant dans le cadre d’une affection digestive sont rares [1,6]. Quelques cas ont été décrits chez des sujets ayant une maladie de Crohn, une maladie cœliaque, un ulcère gastrique, une pancréatite chronique, une cirrhose, un cancer digestif (souvent un lymphome), et chez quelques patients aux antécédents de résection chirurgicale digestive [1,6]. Notre patiente était hospitalisée au début pour une aplasie médullaire sévère d’allure idiopathique. Le retard staturo pondérale, le visage triangulaire avec des taches café au lait et le décès d’un cousin par aplasie à l’âge de 14 ans faisaient évoquer une maladie génétique mais le caryotype était normal. Les polyarthralgies avec tuméfaction des articulations faisaient évoquer une hémarthrose plus qu’une arthrite, les bilans inflammatoire et immunologique étaient négatifs. La ponction de l’articulation n’était pas indiquée vu la thrombopénie et le risque d’insémination infectieuse chez une patiente neutropénique. La recherche des anticorps antiendomesium et anti gliadine devant le syndrome de malabsorption clinique et biologique sévère avec le retard staturo pondérale était négative. Le TCA allongé faisait suspecter la présence d’anticorps anti phospholipide. Cependant, notre patiente était décédée avant d’étiqueter une étiologie causale spécifique.

Le traitement de la maladie gélatineuse repose avant tout sur le traitement de la maladie causale. En cas de cachexie, comme dans notre observation, les lésions médullaires et la pancytopénie régressent souvent après correction de l’état nutritionnel [1,3,8].

## Conclusion

La transformation gélatineuse de la moelle osseuse est une pathologie hématologique rare, se manifestant habituellement par une pancytopénie. Le diagnostic repose sur l’analyse anatomopathologique d’une biopsie ostéomédullaire. Les causes sont variables, mais la transformation gélatineuse de la moelle osseuse est habituellement due à une maladie systémique sévère s’accompagnant d’une cachexie.

## Contribution des auteurs

Les auteurs ont participé a la prise en charge du patient et à la rédaction du manuscrit. Tous les auteurs ont lu et approuvé la version finale du manuscrit.

## Conflits d’intérêts

Les auteurs ne déclarent aucun conflit d’intérêts

## References

[1] Böhm J (2000). Gelatinous transformation of the bone marrow: the spectrum of underlying diseases.. Am J Surg Pathol..

[2] Feugier P, Guerci A, Boman F, Stockemer V, Lederlin P (1995). Transformation gélatineuse de la moelle osseuse: À propos de trois observations.. Rev Med Interne..

[3] Stroup JS, Stephens JR, Baker DL (2007). Gelatinous bone marrow in an HIV-positive patient.. Proc (Bayl Univ Med Cent)..

[4] Seaman JP, Kjeldsberg CR, Linker A (1978). Gelatinous transformation of the bone marrow. Hum Pathol..

[5] Mehta K, Gascon P, Robboy S (1992). The gelatinous bone marrow (serous atrophy) in patients with acquired immunodeficiency syndrome-Evidence of excess sulphated glycosaminoglycan.. Arch Pathol Lab Med..

[6] Boullu-Ciocca S, Darmon P, Sébahoun G, Silaghi A, Dutour-Meyer A (2005). Transformation gélatineuse de la moelle osseuse au cours de l’anorexie mentale.. Annales d’Endocrinologie..

[7] Abella E, Feliu E, Granada I, Millá F, Oriol A, Ribera JM, Sánchez-Planell L, Berga LI, Reverter JC, Rozman C (2002). Bone marrow changes in anorexia nervosa are correlated with the amount of weight loss and not with other clinical findings.. Am J Clin Pathol..

[8] Mokrani N, Bourgeois V, Guernou M, Cobert J, Ait-Mouloud S, Bartoli E, Delcenserie R, Chatelain D (2008). La transformation gélatineuse de la moelle osseuse: Une cause rare de pancytopénie chez un malade cachectique aux antécédents d’oesogastrectomie et de colectomie.. Gastroenterol Clin Biol..

